# Optimizing expression of a single copy transgene in *C. elegans*

**DOI:** 10.17912/micropub.biology.000394

**Published:** 2021-05-12

**Authors:** Scott Dour, Michael Nonet

**Affiliations:** 1 Washington University Medical School, St. Louis, MO USA

## Abstract

The utility of single copy transgenic insertions in *C. elegans* is often limited by low expression. We examined the effects of modifying the trans-splicing signal, the Kozak ribosome binding site, the N-terminal amino acid of the reporter and the 3′ UTR sequences on the expression level of a *mec-4* promoter GFP transgene. The trans-splicing signal and the 3′ UTR had most dramatic effects on expression while modifying the Kozak signal or the N-terminal amino acid had less influence on expression.

**Figure 1. Expression levels of transgenic insertions with altered regulatory elements:**
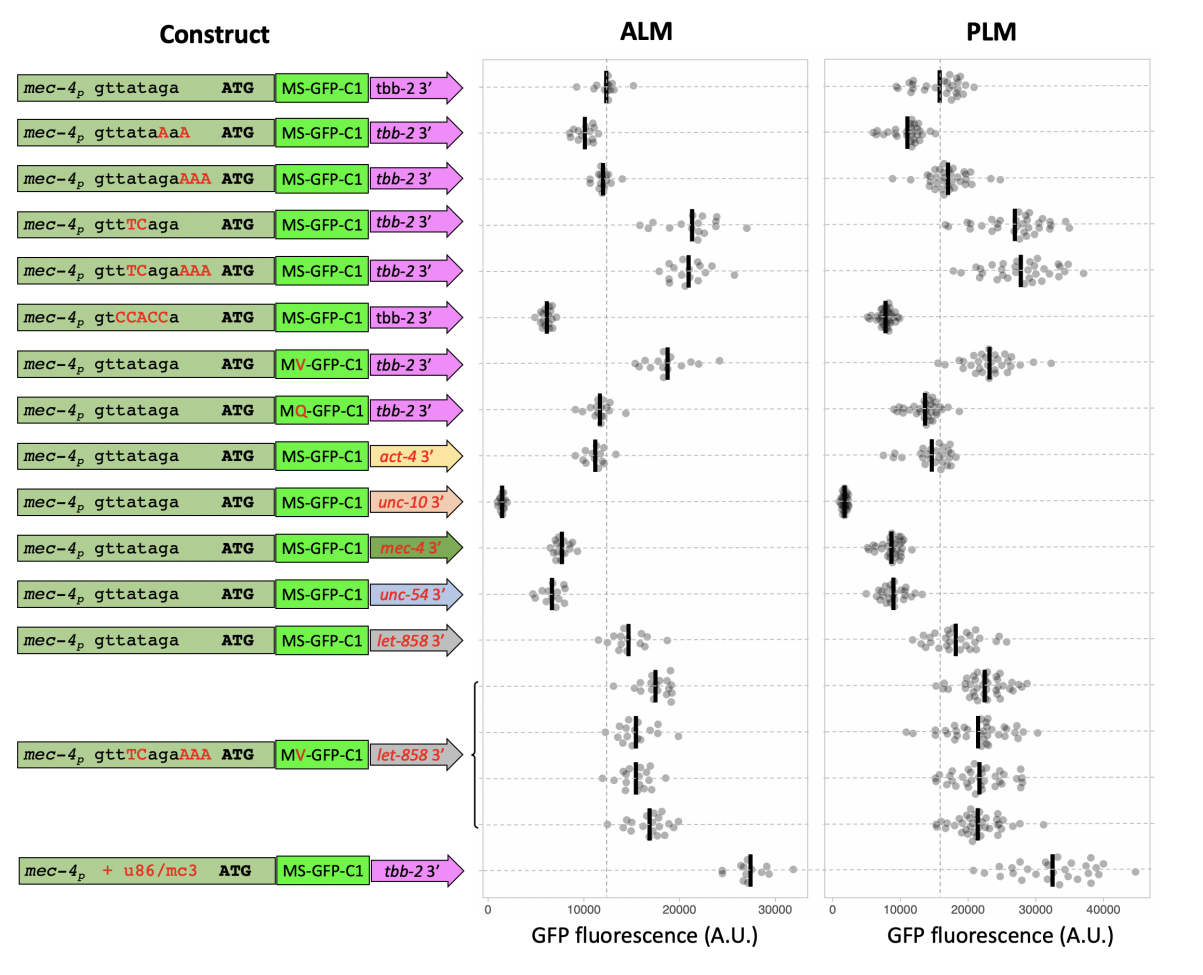
Quantification of GFP expression in ALM and PLM soma of L4 animals with homozygous single copy insertions of constructs schematized on the left. The *mec-4* promoter is shown in olive green, GFP in bright green, and 3’ UTRs in other colors. Modifications to the promoter and GFP are highlighted in red. Individual measurements (filled grey circles) and the mean (black bar) are shown. The units are defined identically for ALM and PLM measurements. See strain list for the exact genotype of animals analyzed. All of the transgenes also express in AVM and PVM. None of the transgenes express in any other cell types to detectable levels. n=13-19 for ALM and 26-39 for PLM.

## Description

Several methods are currently available to create single copy *C. elegans* transgenic animals that express recombinant proteins under the control of specific promoters. Unfortunately, in many cases these transgenic animals express the engineered tools at levels too low to be practically used in routine experiments. We examined the effects of modifications to the trans-splicing sequence, the Kozak ribosomal binding site that promotes translational initiation (Kozak 1987), the N-terminal amino acid controlling N-end rule-mediated protein stability, and the 3′ UTR sequences which regulate message stability on steady state GFP levels in a set of single copy insertions at the same position. Our results document that modifying each of the three components can influence expression levels.

We used an efficient RMCE protocol (Nonet 2020) to create the transgenic animals. Modified versions of a *mec-4* promoter GFP-C1 *tbb-2* 3′ UTR DNA construct were created in an RMCE integration vector using a Golden Gate cloning approach, then integrated using a standard injection protocol. After outcrossing the expression level of GFP at steady state in L4 animal PLM and ALM soma was quantified (Figure 1).

Modification of the sequence upstream of the ATG to contain a computationally determined optimal *C. elegans* consensus Kozak sequence (Blumenthal and Steward 1997) reduced the steady state level of GFP. However, since the replacement also alters the trans-splice acceptor, we also inserted 3 A bases to add a consensus Kozak site without disrupting the splicing signal. This modification had no influence on expression. Modification of the trans-slice acceptor sequence from TTATAG to the consensus TT**TC**AG increased steady state levels ~ two fold, consistent with studies that have shown disruption of the trans-splice signal reduces translation efficiency *in* *vivo* (Yang *et al.* 2017). Disrupting the trans-splice signal by mutating to a non-consensus sequence in the -1 to -5 sites (TCCACC) had an opposing effect reducing expression level about two-fold.

Protein expression levels are also regulated by the N-terminal sequence of proteins, through a biological process known as the N-end rule (Gonda *et al.* 1989). Modification of the first post-Met amino acid of the GFP-C1 protein coding sequence improved expression when the amino acid was changed to valine, the most stabilizing amino acid, and reduced slightly when changed to the unfavorable amino acid glutamine. Although these effects are not dramatic, conformity to the N-end rule is complex depending on additional factors such as inherent structure of the N-terminal region and presence of lysine residues for ubiquitin modification (Varshavsky 2011). Thus, the effects of N -terminal residues may be much more significant for proteins other than GFP.

In addition, it is well documented that expression levels in *C. elegans* are often strongly influenced by 3′ UTR sequences especially in germline tissue (Merritt *et al.* 2008). We replaced the *ttb-2* 3′ UTR with multiple widely used 3′ UTRs as well as the native *mec-4* 3′ UTR and the neuronal *unc-10* 3′ UTR. The effect on GFP levels ranged over 10-fold. *let-858* was the most and *unc-10* the least efficacious 3’ sequence. Note that we used a short *unc-54* 3′ UTR rather than the traditional longer sequence that contains the *aex-5* promoter and often yields posterior intestinal background expression (Silva-García *et al.* 2019). These experiments highlight the robust influence 3′ UTRs have on expression levels. Which 3′ UTRs are most favorable is likely to be cell-type specific, so the same UTRs may not be the most robust in other cell types.

To assess if the effects are additive we created a transgene that incorporated the most effective trans-splicing and protein stability signals and the most optimal 3’ UTR. Disappointingly the multi-mutant construct expressed less strongly than the individual modified promoters, indicating that the elements interact with each other in complex ways to determine the overall expression level. Since this result was unexpected, we quantified the expression levels of 4 independently isolated identical insertions of the multi-mutant construct, and all behaved very similarly. This supports our prior experience that the *jsTi1453* landing site is not significantly influenced by epigenetic factors under standard laboratory conditions.

Finally, we manipulated the *mec-4* promoter in a fashion that is unlikely to be easily performed for most promoters. Extensive analysis of the *mec-3*, *mec-4* and *mec-7* promoters as well as other *mec-3/unc-86* regulated genes (Xue *et al.* 1992; Duggan *et al.* 1998; Zhang *et al.* 2002) has defined a consensus binding site for the critical UNC-86/MEC-3 transcription factor heterodimer [CATN_(3-4)_AAATGCAT]. The *mec-4* promoter is known to contain one such sequence; CATtatAAATGTAT. We inserted an additional binding site 100 bp upstream of the known binding site by introducing 27 bp that contain CATaagAAATGTAT – an identical sequence to the native binding site in the *mec-4* promoter at the critical bases (capitals). Introduction of this sequence increased expression over two-fold compared to the native promoter and was the most potent of the manipulations performed.

While our studies identify some modifications that can be introduced into transgenic constructs to increase expression, they do not define a clear set of rules that can be implemented to insure high expression. Nevertheless, the simplicity of RMCE integration should make altering transgenic constructs a more realistic option to attempt before resorting to creating multicopy integrated transgenes.

## Methods

**Methods**

*C. elegans* was maintained on NGM agar plates spotted with OP50 at 22.5°C or at 25°C during the RMCE protocol.

**RMCE transgenesis**

Inserts were cloned into pLF3FShC (Addgene # 153083; Nonet, 2020) and injected at ~50 ng/µl into *jsTi1453; bqSi711* young adults. Integrants were identified and isolated as described in detail in Nonet (2020). Single copy insertions were outcrossed to *jsTi1453; him-8(e1489)* to create *jsTi1453 jsSi#; him-8(e1489)* strains.

**Microscopy**

For quantification of GFP signals, homozygous L4 hermaphrodite animals were mounted on 2% agar pads in a 2 µl drop of 1mM levamisole in phosphate buffered saline and imaged on an Olympus (Center Valley, PA) BX-60 microscope equipped with a Qimaging (Surrey, BC Canada) Retiga EXi monochrome CCD camera, a Lumencor AURA LED light source, Semrock (Rochester, NY) GFP-3035B and mCherry-A-000 filter sets, and a Tofra (Palo Alto, CA) focus drive, run using micro-manager 2.0ß software (Edelstein *et al.* 2014) using a 40X air lens at 20% LED power with 200 ms exposures. PLM soma and ALM soma signals were quantified using the FIJI version of ImageJ software (Schindelin *et al.* 2012) as described in Nonet (2020).

**Plasmid constructions**

Modified versions of the NM3732 pLF3FShC *mec-4p* GFP-C1 *tbb-2* plasmid were performed by SapI Golden Gate (GG) assembly inserting modified components from DR274 insert constructs as outlined below. DR274 entry vectors were created by inserting PCR fragments into the vectors using a BsaI GG reaction. Assembly reactions were performed as described in Nonet (2020).

The following were used:

pDD372 GFP-C1 (Dickinson *et al.* 2018)

NMp3469 DR274 FP-BsaI (Nonet, 2020)

NMp3643 pLF3FShC (Nonet, 2020)

NMp3694 DR274 AAG GTA tbb-2 3′ UTR (Nonet, 2020)

NMp3702 DR274 AAG GTA-BsaI (Nonet, 2020)

NM93732 pLF3FShC mec-4p GFP-C1 tbb-2 3’ UTR (Nonet, 2020)

NMp3751 DR274 AAG GTA act-4 3′ UTR (Nonet, 2020)

NMp3758 DR274 AAG GTA mec-4 3′ UTR

The *mec-4* 3’ UTR was amplified from N2 genomic DNA using oligonucleotides NMo6654/6655 and inserted into NMp3702.

NMp3759 DR274 AAG GTA unc-10 3′ UTR

The *unc-10* 3’ UTR was amplified from N2 genomic DNA using oligonucleotides NMo6656/6657 and inserted into NMp3702.

NMp3760 DR274 AAG GTA unc-54 3′ UTR

The *unc-54* 3’ UTR was amplified from N2 genomic DNA using oligonucleotides NMo6658/6659 and inserted into NMp3702.

NMp3761 DR274 FP (MV)GFP C1

GFP-C1 was amplified from pDD372 using NMo6660/6662 and inserted into NMp3469.

NMp3762 DR274 FP (MQ)GFP C1

GFP-C1 was amplified from pDD372 using NMo6661/6662 and inserted into NMp3469.

NMp3763 DR274 TGG ATG mec-4p

The *mec-4* promoter was amplified from N2 genomic DNA using oligonucleotides NMo6663/6664 and inserted into NMp3698.

NMp3764 DR274 TGG ATG mec-4p wKr

The *mec-4* promoter was amplified from N2 genomic DNA using oligonucleotides NMo6663/6665 and inserted into NMp3698.

NMp3765 DR274 TGG ATG mec-4p TS

The *mec-4* promoter was amplified from N2 genomic DNA using oligonucleotides NMo6663/6667 and inserted into NMp3698.

NMp3766 DR274 AAG GTA let-858 3′ UTR

The let-858 3’ UTR was amplified from N2 genomic DNA using oligonucleotides NMo6656/6657and inserted into NMp3702.

NMp3778 DR274 TGG ATG mec-4p TS(-)

The *mec-4* promoter was amplified from N2 genomic DNA using oligonucleotides NMo6663/6666 and inserted into NMp3698.

NMp3779 pLF3FShC mec-4p (MV)GFP-C1

The *mec-4* promoter from NMp3763, *GFP-C1(MV)* from NMp3761, and the *tbb-2* 3’ UTR from NMp3694 were co-assembled into NMp3643.

NMp3780 pLF3FShC mec-4p (MQ)GFP-C1

The *mec-4* promoter from NMp3763, *GFP-C1(MQ)* from NMp3762, and the *tbb-2* 3’ UTR from NMp3694 were co-assembled into NMp3643.

NMp3781 pLF3FShC mec-4p GFP-C1 act-4 3′

The *mec-4* promoter from NMp3763, *GFP-C1* from pDD372, and the *act-4* 3’ UTR from NMp3751 were co-assembled into NMp3643.

NMp3782 pLF3FShC mec-4p GFP-C1 let-858 3′

The *mec-4* promoter from NMp3763, *GFP-C1* from pDD372, and the *let-858* 3’ UTR from NMp3766 were co-assembled into NMp3643.

NMp3785 pLF3FShC mec-4p GFP-C1 mec-4 3′

The *mec-4* promoter from NMp3763, *GFP-C1* from pDD372, and the *mec-4* 3’ UTR from NMp3758 were co-assembled into NMp3643.

NMp3786 pLF3FShC mec-4p GFP-C1 unc-10 3′

The *mec-4* promoter from NMp3763, *GFP-C1* from pDD372, and the *unc-10* 3’ UTR from NMp3759 were co-assembled into NMp3643.

NMp3787 pLF3FShC mec-4p GFP-C1 unc-54 3′

The *mec-4* promoter from NMp3763, *GFP-C1* from pDD372, and the *unc-54* 3’ UTR from NMp3760 were co-assembled into NMp364.

NMp3788 pLF3FShC mec-4p wKr GFP-C1

The *mec-4* worm Kozak replacement promoter from NMp3764, *GFP-C1* from pDD372, and the *tbb-2* 3’ UTR from NMp3694 were co-assembled into NMp3643

NMp3789 pLF3FShC mec-4p TS GFP-C1

The *mec-4* optimized trans-splice promoter from NMp3765, *GFP-C1* from pDD372, and the *tbb-2* 3’ UTR from NMp3694 were co-assembled into NMp3643.

NMp3790 pLF3FShC-mec-4p TS(-) GFP-C1

The *mec-4* trans-splice signal lesioned promoter from NMp3778, *GFP-C1* from pDD372, and the *tbb-2* 3’ UTR from NMp3694 were co-assembled into NMp3643.

NMp3824 pLF3FShC mec-4(+u86m3)p GFP-C1

MNp3732 was amplified with oligonucleotides NMo6742/6743, kinased and religated. NMp4000 DR274 TGG ATG mec-4p TS wK+

The *mec-4* promoter was amplified from NMp3779 and using oligonucleotides NMo6663/7007 and inserted into NMp3698.

NMp4001 DR274 TGG ATG mec-4p wK+

The *mec-4* promoter was amplified from NMp3779 and using oligonucleotides NMo6663/7028 and inserted into NMp3698.

NMp4009 pLF3FShC mec-4p TS wK+ GFP-C1

The optimized *mec-4* promoter from NMp4000, *GFP-C1* from pDD372, and the *tbb-2* 3’ UTR from NMp3694 were co-assembled into NMp3643.

NMp4010 pLF3FShC mec-4p wK+ GFP-C1

The optimized Kozak site *mec-4* promoter from NMp4001, *GFP-C1* from pDD372, and the *tbb-2* 3’ UTR from NMp3694 were co-assembled into NMp3643.

NMp4020 pLF3FShC mec-4p TS wK+ (MV)GFP-C1 let-858

The optimized *mec-4* promoter from NMp4000, *(MV)GFP-C1* from NMp3761, and the *let-858* 3’ UTR from NMp3782 were co-assembled into NMp3643.

**Oligonucleotides**

**Table:** 

**NMo number**	**Sequence**
6652	ttGGTCTCAgAAGTGAattttcaaattttaaatactgaatatttg
6653	gcGGTCTCTcTACccaagcgaggacaattct
6654	ttGGTCTCAgAAGTGAatttgttttttcttgttttaaagtt
6655	gcGGTCTCTcTACgcagcttacagtatctttgtatt
6656	ttGGTCTCAgAAGTAAcaaatttcatatgtttttgtttgtt
6657	gcGGTCTCTcTACcattctccgttttctattgagt
6658	ttGGTCTCAgAAGTGAAGCTCCGCATCGG
6659	gcGGTCTCTcTACgtcataaactgaaacgtaacatatg
6660	ttGGTCTCAgATGGTCAGTAAAGGAGAAGAATTGTTCACT
6661	ttGGTCTCAgATGCAGAGTAAAGGAGAAGAATTGTTCACT
6662	gcGGTCTCTcCTTGTAGAGCTCGTCCATT
6663	ttGGTCTCAgTGGggttccggagcagttc
6664	ttGGTCTCTtCATtctataacttgatagcgataa
6665	ttGGTCTCTtCATttttataacttgatagcgataaaaaaaatagc
6666	ttGGTCTCTtCATtggtggacttgatagcgataaaaaaaatagc
6667	ttGGTCTCTtCATtctgaaacttgatagcgataaaaaaaatagc
6742	GAAATGTATAGAATACCAGTGCCTGGTGTTTGAGATGTTCTG
6743	TTATGATCCATTTCAACACACTTTCATGGATCTTATCTTGC
7007	TTGGTCTCTTCATTTTTCTGAAACTTGATAGCGAT
7028	TTGGTCTCTTCATTTTtctataacttgatagcgataaa

## Reagents

**Worm Strains**

**Table:** 

**NM strain**	**Genotype**	**Source**
NM5161	*jsTi1453 I; bqSi711 IV*	Nonet, 2020; CGC
NM5187	*jsTi1453 I; him-8(e1489) IV*	Nonet, 2020; CGC
NM5209	*jsTi1453 jsSi1514 [mec-4p GFP-C1 tbb-2 3’] I; him-8(e1489) IV*	Nonet, 2020; CGC
NM5285	*jsTi1453 jsSi1556 [mec-4p (MV)GFP-C1 tbb-2 3’] I; him-8(e1489) IV*	This work
NM5286	*jsTi1453 jsSi1557 [mec-4p (MQ)GFP-C1 tbb-2 3’] I; him-8(e1489) IV*	This work
NM5287	*jsTi1453 jsSi1558 [mec-4p GFP-C1 unc-10 3’] I; him-8(e1489) IV*	This work
NM5288	*jsTi1453 jsSi1559 [mec-4p GFP-C1 act-4 3’] I; him-8(e1489) IV*	This work
NM5289	*jsTi1453 jsSi1561 [mec-4p GFP-C1 mec-4 3’] I; him-8(e1489) IV*	This work
NM5328	*jsTi1453 jsSi1562 [mec-4p TS(-) GFP-C1 tbb-2 3’] I; him-8(e1489) IV*	This work
NM5329	*jsTi1453 jsSi1563 [mec-4p TS GFP-C1 tbb-2 3’] I; him-8(e1489) IV*	This work
NM5330	*jsTi1453 jsSi1564 [mec-4p wKr GFP-C1 tbb-2 3’] I; him-8(e1489) IV*	This work
NM5331	*jsTi1453 jsSi1566 [mec-4p GFP-C1 unc-54 3’] I; him-8(e1489) IV*	This work
NM5332	*jsTi1453 jsSi1573 [mec-4p GFP-C1 let-858 3’] I; him-8(e1489) IV*	This work
NM5333	*jsTi1453 jsSi1573 [mec-4(XB)p GFP-C1 tbb-2 3’] I; him-8(e1489) IV*	This work
NM5436	*jsTi1453 jsSi1636 [mec-4p wK+ GFP-C1 mec-4 3’] I; him-8(e1489) IV*	This work
NM5438	*jsTi1453 jsSi1637 [mec-4p TS wK+ GFP-C1 mec-4 3’] I; him-8(e1489) IV*	This work
NM5441	*jsTi1453 jsSi1642 [mec-4p TS wK+ (MV)GFP-C1 let-858 3’] I; bqSi71 IV*	This work
NM5450	*jsTi1453 jsSi1650 [mec-4p TS wK+ (MV)GFP-C1 let-858 3’] I; bqSi711 IV*	This work
NM5451	*jsTi1453 jsSi1651 [mec-4p TS wK+ (MV)GFP-C1 let-858 3’] I; bqSi711 IV*	This work
NM5452	*jsTi1453 jsSi1652 [mec-4p TS wK+ (MV)GFP-C1 let-858 3’] I; bqSi711 IV*	This work

**Reagents**

Plasmids and worm strains are available by request from MLN and will be submitted to Addgene and the *Caenorhabditis* Genetics Center if demand levels warrant it.
